# Interactive Effects of Glycine Equivalent, Cysteine, and Choline on Growth Performance, Nitrogen Excretion Characteristics, and Plasma Metabolites of Broiler Chickens Using Neural Networks Optimized with Genetic Algorithms

**DOI:** 10.3390/ani10081392

**Published:** 2020-08-11

**Authors:** Philipp Hofmann, Wolfgang Siegert, Hamed Ahmadi, Jochen Krieg, Moritz Novotny, Victor D. Naranjo, Markus Rodehutscord

**Affiliations:** 1 Institute of Animal Science, University of Hohenheim, 70599 Stuttgart, Germany; philipp_hofmann@uni-hohenheim.de (P.H.); jochen.krieg@uni-hohenheim.de (J.K.); moritz.novotny@uni-hohenheim.de (M.N.); markus.rodehutscord@uni-hohenheim.de (M.R.); 2 Bioscience and Agriculture Modeling Research Unit, Department of Poultry Science, Tarbiat Modares University, Tehran 14115-336, Iran; hamed.ahmadi@modares.ac.ir; 3 Evonik Nutrition & Care GmbH, 63457 Hanau, Germany; victor.naranjo@evonik.com

**Keywords:** broiler chickens, interactive effect, glycine equivalent, cysteine, choline, neural networks, nitrogen-utilization efficiency, uric acid, ammonia

## Abstract

**Simple Summary:**

The negative effects of nitrogen emissions caused by animal husbandry on the environment can be reduced by lowering the crude protein content in the diets of broiler chickens. The nonessential amino acids glycine and serine, investigated together as glycine equivalent, can limit the growth of broiler chickens fed diets that are low in crude protein. The response of broiler chickens to dietary glycine equivalent is not constant and is affected by endogenous precursors of glycine equivalent and metabolic processes that dissipate glycine equivalent. Choline can be converted to glycine, and glycine equivalent is required to form cysteine from methionine. The present study investigated interactive effects among dietary glycine equivalent, cysteine, and choline in broiler chickens. The results showed that the gain:feed ratio increased with dietary glycine equivalent supplementation. The extent of interactive effects among glycine equivalent, cysteine, and choline on the gain:feed ratio was hardly pronounced. Very high nitrogen-utilization efficiency with low variation among treatments was found. The findings indicate that small differences in nitrogen-utilization efficiency caused low glycine equivalent dissipation for nitrogen excretion, likely resulting in small interactive effects among dietary glycine equivalent, cysteine, and choline. These results contribute to further dietary crude protein reduction in feed for broiler chickens.

**Abstract:**

Responses of broiler chickens to dietary glycine equivalent (Gly_equi_) are affected by dietary cysteine and choline. Hence, this study investigated interactive effects among dietary Gly_equi_, cysteine, and choline on the growth of broiler chickens. Male Ross 308 broiler chickens were maintained in 105 metabolism units (10 birds/unit) from days 7 to 22. Excreta were collected in 12-h intervals from days 18 to 21. Blood was sampled on day 22 (1 bird/unit). Five levels each of Gly_equi_ (9–21 g/kg), cysteine (2–5 g/kg), and choline (0.5–1.7 g/kg) were tested under 15 diets in 7 replicates each following a fractional central composite design. Another diet was provided to five metabolism units (15 birds/unit) to measure prececal amino acid digestibility. Data were evaluated using neural networks. The gain:feed ratio (G:F) increased with digestible Gly_equi_ intake. Differences between low and high digestible cysteine intake were low. Effects of choline intake on G:F were low. Nitrogen-utilization efficiency (NUE) was high (≥77%), with low variation among treatments. Plasma metabolites varied among treatments and indicated that metabolism of Gly_equi_, cysteine, and choline was influenced. These findings showed that interactive effects of dietary Gly_equi_, cysteine, and choline on growth were small, possibly because NUE was barely influenced.

## 1. Introduction

The negative impacts of nitrogen (N) emissions from animal husbandry can be diminished by reducing dietary crude protein (CP) in feeds. Very low CP diets are often accompanied by decreased growth in broiler chickens when the nonessential amino acids (AA) glycine (Gly) and serine (Ser)—considered together as glycine equivalent (Gly_equi_)—are not adequately supplied because these AA can induce a growth limiting effect [1,2,3]. The Gly_equi_ requirement is not constant, with dietary Gly_equi_ concentrations varying from below 10 to above 16 g/kg among studies to achieve 95% of the maximum gain:feed ratio (G:F) [4]. These variations were explained by different amounts of Gly_equi_ being dissipated for metabolic processes and by varying dietary concentrations of endogenous precursors of Gly and Ser.

One metabolic process for which Gly_equi_ is required is the endogenous formation of cysteine (Cys) from methionine (Met). Homocysteine (Hcy) and cystathionine (Cysta) are intermediates in this metabolic conversion, and Ser is required to form Cysta from Hcy [5]. This was suggested as one explanation for the interactive effects between dietary Gly_equi_ and Cys on growth found in a previous meta-analysis [6]. The extent of these interactive effects cannot be explained only by the formation of Cys from Met. Further influences must have been responsible for the extent of interactive effects between dietary Gly_equi_ and Cys on growth found in the meta-analysis by Siegert et al. [6].

Choline (Cho) is an endogenous precursor of Gly in the metabolic pathway involving the conversion of Cho → betaine → dimethylglycine → sarcosine → Gly [7]. The conversion of Cho to Gly is connected to Cys formation from Met and can only occur when one methyl group of betaine is transferred to Hcy by betaine-Hcy-methyltransferase (BHMT; EC 2.1.1.5) to form Met [8]. Concurrently, Met can be synthesized by transferring the methyl groups of dimethylglycine and sarcosine via the folate pool to Hcy by Met-synthase (MS; EC 2.1.1.13) [9]. Interactive effects between Gly_equi_ and Cho on growth have been described previously; however, the extent of these interactive effects cannot alone be explained by the endogenous conversion of Cho to Gly [10]. Therefore, varying dietary Cys concentrations that affect the conversion of Hcy to Met could influence the extent of interactive effects between Gly_equi_ and Cho on growth. Further, Cho as a methyl group donor and precursor of Gly might provide a further explanation regarding the interactive effects of Gly_equi_ and Cys on growth as observed in the meta-analysis by Siegert et al. [6].

The formation of uric acid (UA) is a Gly_equi_-dissipating process because one molecule of Gly is required to form the purine ring of UA [11]. An increase in nitrogen-utilization efficiency (NUE) leads to less urinary UA-N excretion, resulting in decreased Gly_equi_ requirement with increasing NUE [4]. Therefore, varying NUE influences the response to dietary Gly_equi_ and likely affects the interactive effects among Gly_equi_, Cys, and Cho on growth.

We hypothesized that investigating different dietary concentrations of Gly_equi_, Cys, and Cho together could explain the interactive effects between Gly_equi_ and Cys and between Gly_equi_ and Cho that have previously been found in other studies. Therefore, we investigated the interactive effects among dietary Gly_equi_, Cys, and Cho in a growth study. Digestibility was analyzed to determine the prececal digestible AA intake. Total N excretion was measured to determine NUE, and plasma metabolites were analyzed as indicators of metabolism of Gly_equi_, Cys, and Cho. Neural networks optimized with genetic algorithms were used for data evaluation.

## 2. Materials and Methods

### 2.1. Experimental Design

Two trials were run simultaneously. All dietary treatments were randomly distributed in a completely randomized block design.

A growth trial was conducted to investigate the interactive effects among dietary Gly_equi_, Cys, and Cho at five concentrations on growth, total N excretion, and plasma metabolites. A fractional central composite design [12] was used. This resulted in 15 dietary treatments that were tested in seven replicates each.

A digestibility trial was undertaken to determine the prececal AA digestibility of one diet in five replicates. This was done to obtain the prececal digestible AA concentrations of diets used in the growth trial.

### 2.2. Birds and Housing

The experiment was performed at the Agricultural Experiment Station of the University of Hohenheim, Germany. It was approved by the Regierungspräsidium Tübingen, Germany (Project no. HOH 48/17 TE), and was conducted according to the German Animal Welfare Legislation. Male Ross 308 broiler hatchlings were delivered by a commercial hatchery (Brüterei Süd ZN der BWE-Brüterei Weser-Ems GmbH & Co. KG, Regenstauf, Germany). Birds were held in five floor pens (3 m × 4 m) on dedusted wood shavings and received a commercial starter diet containing 215 g CP/kg and 12.5 MJ ME/kg (415002025 Club Mastkükenstarter, Deutsche Tiernahrung Cremer GmbH & Co. KG, Mannheim, Germany) up to day 7. Then, 1125 birds were distributed to metabolism units and placed on a mesh-wired floor. The birds were allocated to the metabolism units so that an equal mean bird weight was achieved in every unit of both the trials. Feed and water were provided for ad libitum consumption throughout the experiment. Lighting was continuous during the first 3 days after placement, followed by 18-h light and 6-h dark cycle until the end of the experiment. Temperature was maintained at 34 °C for the first 3 days and was gradually decreased to 26 °C on the last day of the experiment.

In the growth trial, a total of 1050 broiler chickens were distributed to 105 (1 m × 1 m × 1 m) metabolism units. Each diet was tested in 7 metabolism units and each metabolism unit contained 10 birds. The experimental diets were fed from days 7 to 22.

In the digestibility trial, 15 out of 75 birds were assigned to one of five (2 m × 1 m × 1 m) metabolism units. The birds received the experimental diets from days 7 to 21.

### 2.3. Experimental Diets

The diets were mainly based on corn and casein (Table 1) and were mixed at the certified feed mill of the University of Hohenheim. Concentrations of all nutrients listed herein are based on a standardized dry matter (DM) of 88%, unless otherwise stated. The concentrations of essential AA including Met + Cys were calculated at 105% of the recommendations of the Gesellschaft für Ernährungsphysiologie [13]. The concentrations of the nonessential AA alanine, aspartic acid/asparagine, and glutamic acid/glutamine were calculated at 8.6, 15.4, and 28.1 g/kg, respectively, because lower concentrations of these AA cause limited growth [14]. Concentrations of AA were adjusted by adding AA in free form. Other nutrients were formulated to meet or exceed the recommendations of the Gesellschaft für Ernährungsphysiologie [13].

In the growth trial, different concentrations of dietary Gly_equi_ (9, 12, 15, 18, and 21 g/kg), Cys (2.0, 2.75, 3.5, 4.25, and 5.0 g/kg), and Cho (0.5, 0.8, 1.1, 1.4, and 1.7 g/kg) (Table 2) were obtained by adding free Gly, free l-Cys·HCl·H_2_O, and choline chloride in variable proportions to a basal mixture. Free dl-Met inclusions were altered to ensure constant Met + Cys concentrations among treatments. Cornstarch was used to compensate for mass differences. Gly_equi_ concentrations used in this study were similar to those that were shown to influence growth in the literature [4]. Cho concentrations were calculated according to those reported by Siegert et al. [10], and Cys concentrations were calculated to include the range of Cys concentrations reported in the meta-analysis by Siegert et al. [6].

The diet fed in the digestibility trial was mixed using the same basal mixture utilized for the growth trial. The concentrations of dietary Gly_equi_, Cys, and Met were equal to the lowest concentrations of these nutrients in the growth trial. TiO_2_ was added as an indigestible marker.

One basal mixture that represented approximately 98% of the final diets of both trials was established, divided into 16 parts, and complemented with the ingredients required for the final diets. The diets were pelleted without using steam through a 3-mm die. The calculated AA concentrations were confirmed by analyses. The variation of the analyzed AA (apart from Gly_equi_, Cys, and Met) was low among the treatments (Table 2 and Table S1). Dietary CP ranged between 172 and 185 g/kg. The analyzed Cho concentrations were within the 15% range of measurement accuracy, thus confirming the calculated values. Calculated dietary Cho concentrations were used for subsequent calculations.

### 2.4. Experimental Procedures

In the growth trial, total bird weight and feed consumption in each metabolism unit were recorded on days 7, 18, and 21 of the experiment to determine the mortality-corrected average daily gain (ADG), average daily feed intake (ADFI), and G:F. Feed intake was measured on a DM basis by determining the DM content of each diet on the same days. Total excreta were collected at 12-h intervals from days 18 to 21 of the experiment. Excreta were carefully cleaned to remove feathers, skin scales, and spilled feed pellets before each collection and then immediately stored at −20 °C. Spilled feed was picked up throughout the experiment, weighed after being dried, and used for correction of feed DM intake. The excreta of birds collected in four metabolism units were not used for data analysis because of errors during excreta collection. The birds were examined at least twice daily. In the case of death, dead birds were considered in the calculation of ADG, ADFI, and G:F. Blood was sampled on day 22 from the brachial vein of one bird per metabolism unit and was placed into a 4 mL heparinized tube. The bird whose body weight was the closest to the mean body weight of birds in each unit was selected for blood sampling. The feeding regime according to Donsbough et al. [15] described in detail by Hofmann et al. [14] was used before blood sampling. The sample was immediately centrifuged at 1500× *g* for 10 min at 4 °C, and the resulting blood plasma was frozen at −20 °C.

In the digestibility trial, birds were fed the diet with medium concentrations of Gly_equi_, Cys, Met, and Cho (treatment H in the growth trial) from days 7 to 16 to ensure similar physiological development of the birds in the two trials. Subsequently, they received diet P (with the lowest concentrations of Gly_equi_, Cys, and Met). On day 21, the birds were stunned with a gas mixture of 35% CO_2_, 35% N_2_, and 30% O_2_ and euthanized with CO_2_ exposure. Digesta was collected from the distal two-thirds of the section between diverticulum vitellinum and 2 cm anterior to the ileo-ceco-colonic junction by flushing with cold, double-distilled water. Digesta of the 15 birds in each metabolism unit was pooled and immediately frozen at −20 °C.

### 2.5. Chemical Analyses

Diet H was ground using a centrifugal mill (ZM 200; Retsch GmbH, Haan, Germany) through a 0.5 mm sieve to analyze it for crude ash (method no. 8.1), crude fat (method no. 5.1.1), crude fiber (method no. 6.1.1), DM (method no. 3.1), and starch (method no. 7.2.1) according to the official methods for nutrient analyses in Germany [16]. A vibrating cup mill (Pulverisette 9; Fritsch GmbH, Idar−Oberstein, Germany) was used to pulverize all diets and freeze−dried digesta and excreta for AA, CP, Ti, and UA analyses. CP concentration was determined using the Dumas method. The official method 994.12 of the Association of Official Analytical Chemists [17] was used for AA analysis, with minor laboratory adaptions described by Siegert et al. [10]. CP and AA were analyzed by Evonik Nutrition & Care GmbH (Hanau, Germany). Ti was analyzed as described previously [18]. Cho was analyzed using the method outlined by Siegert et al. [10] by LUFA−ITL GmbH (Kiel, Germany). At least one diet per Cho concentration was analyzed to confirm the values calculated during diet formulation. Accurate quantification of Cho concentrations in digesta was not possible with a limit of quantification of 0.2 g/kg DM; therefore, the digestibility of Cho was not determined. The preparation of excreta samples and determination of DM, N, ammonia (NH_3_), and UA in the excreta were conducted according to the method described by Hofmann et al. [14]. One milliliter of each blood plasma sample was freeze-dried before being analyzed for free AA and biogenic amines by Evonik Nutrition & Care GmbH (Hanau, Germany). Freeze-dried blood plasma was resolubilized with lithium citrate buffer solution and peptides and proteins were precipitated using sulfosalicylic acid. AA were separated using the Biochrom 30 AA analyzer (Biochrom Ltd., Cambridge, UK). The separated AA were then mixed with ninhydrin and quantified photometrically. Blood plasma biogenic amines were determined using high-performance liquid chromatography with fluorescence detection after pre-column derivatization with dansyl chloride and liquid−liquid extraction with methyl tert-butyl ether. Measured homocystine (Hcys) was used to conclude on Hcy concentrations.

### 2.6. Calculations

The following equations were used to calculate N accretion and NUE:

N accretion (g/d) = N intake (g/day) − total N excretion (g/day) (1)

NUE (%) = N accretion (g/day)/N intake (g/day) × 100 (2)

The uric acid-nitrogen/(uric acid-nitrogen + ammonia-nitrogen) [UA-N/(UA-N + NH_3_-N)] ratio in the excreta was calculated because it has been reported to be suitable for interpreting the physiological processes of N metabolism in broiler chickens [14,19]. Prececal AA digestibility was calculated using the following equation:

Prececal AA digestibility (%) = 100 − [(TiO_2Diet_ × AA_Digesta_)/(TiO_2Digesta_ × AA_Diet_)] × 100 (3)

where, AA_Diet_ and AA_Digesta_ are the concentrations of the individual AA and TiO_2Diet_ and TiO_2Digesta_ are the TiO_2_ concentrations in the diet and the digesta, respectively. Concentrations of prececal digestible Gly_equi_ (dGly_equi_), Cys (dCys), and Met (dMet) in the growth trial diets were determined using digestible AA concentrations determined in the digestibility trial and assuming complete digestibility of free AA. The daily intake of dGly_equi_, dCys, dMet, or Cho was measured for each metabolism unit by multiplying the concentrations of dGly_equi_, dCys, dMet, or Cho with the respective ADFI.

### 2.7. Data Modeling and Optimization

Supervised, fully connected, feed forward neural networks optimized using genetic algorithms were used for data modeling and optimization using MatLab R2017b (The MathWorks Inc., Natick, MA, USA). Genetic algorithms were used to optimize the neural networks by modifying the number of neurons and their coefficients in the hidden layer to minimize mean squared error (MSE). The training of neural networks initiated with one neuron in the hidden layer. The parameters specifying the genetic algorithm [20] were as follows: initial population of 50, generation number of 1000, mutation rate of 0.1, and crossover rate of 0.85. The roulette wheel selection method was used for selecting the elite populations for crossover. An MSE of 0.001 was used as the performance function. Hyperbolic tangent and linear functions were the transfer functions for the hidden and output layers, respectively [21]. The dataset was randomly divided into 70% training and 30% testing set for each model. Independent models were developed for each response trait. The evaluation of model performance was based on the accuracy of model predictions in the training, testing, and entire data sets using the coefficient of determination (R^2^) and root MSE. The intake of dGly_equi_, dCys, and Cho were the independent input variables used for data evaluation because nutrient intake accurately describes the nutrient supply of the birds. The relevance of an input variable to the model performance was determined using the variable total effect (VTE) [22], which represents the contribution of an input variable to the variance of a model output. Higher VTE values indicate a higher influence of input variables on model performance. In addition to defining neural networks, genetic algorithms were utilized to analyze the developed neural network models to find combinations of dGly_equi_, dCys, and Cho intake for maximized and minimized responses. Regression analyses were performed to determine the dietary concentrations corresponding to the respective intake of digestible nutrients using GraphPad Prism 5.0 (GraphPad Software Inc., San Diego, CA, USA). Figures were prepared using the same software. According to the fractional composite design, not all level combinations can be shown without extrapolating, and the range where interactions can be interpreted decreased with the deviance from level 0. Therefore, levels −1, 0, and +1 were defined as low, medium, and high dCys and Cho intake levels, and ranges of dGly_equi_ intake are presented to be wider than high and low dCys and Cho intake. We decided that the relationships between the measured traits and varying nutrients were too low and were not further described when R^2^ ≤ 0.50.

## 3. Results

The initial bird weights among metabolism units ranged between 180 and 191 (standard deviation [SD] 2.7) g/bird on day 7 and were not significantly different among treatments (*p* = 0.885). The survival rate during the experimental phase was ≥ 96% and was independent of treatment (27 out of 1050 birds died in 11 different treatments in the growth trial and 3 out of 75 birds died in the digestibility trial).

### 3.1. Neural Network Model Characteristics

The characteristics of models for traits with R^2^ > 0.50 that were used for further interpretations are described in Table 3 and Table 4, and the corresponding model equations are shown in Table S2. The descriptions of neural network models for traits with R^2^ ≤ 0.50 are shown in Table S3. Maximum output values were determined at extrapolated dGly_equi_, dCys, and Cho intake levels for all traits. Hence, these combinations are not presented.

### 3.2. Prececal Amino Acid Digestibility and Prececal Digestible Nutrient Intake

**Figure 1:**
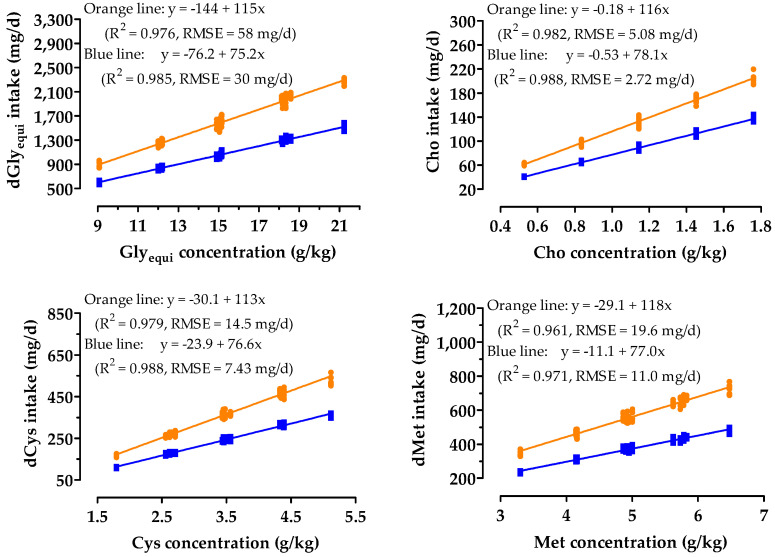
Relationship between diet concentrations and prececal digestible intake of glycine equivalent (dGly_equi_), cysteine (dCys), methionine (dMet), and total choline (Cho) by broiler chickens from days 7 to 21 (blue lines) and days 18 to 21 (orange lines). y is the intake (mg/d) of dGly_equi_, dCys or dMet or the total intake of Cho, and x is the dietary concentration (g/kg) of glycine equivalent (Gly_equi_), cysteine (Cys), methionine (Met), or Cho.

Prececal AA digestibility in the digestibility trial was between 81% for Cys and 96% for arginine and was higher than 90% for most AA (Table 5). Prececal digestibility of Gly, Ser, Cys, and Met was 84%, 84%, 81%, and 95%, respectively. The regression equations used to calculate the dietary concentrations from the intake of digestible nutrients were linear (Figure 1).

### 3.3. Global Sensitivity Analysis

Results of the global sensitivity analysis indicating the importance of independent variables (Table 6) were ranked via the intake of dGly_equi_ > dCys > Cho for G:F, NUE, UA excretion, UA-N/(UA-N + NH_3_-N) ratio, and plasma Gly concentration, the intake of dCys > Cho > dGly_equi_ for NH_3_ excretion and plasma Met, Hcys, and Cysta concentrations, and the intake of dGly_equi_ > Cho > dCys for total N excretion.

### 3.4. Average Daily Gain and Average Daily Feed Intake

Neural networks showed strong relationships among the nutrients and ADG and ADFI (R^2^ ≥ 0.93), and variation in these traits was low (57.0–68.7 g/bird for ADG and 72.7–82.9 g/bird for ADFI). However, the modeled curve shapes of these traits were undulated (Figures S1 and S2) and, therefore, biologically implausible. Similar high R^2^ and undulated curve shapes were obtained when neural networks with a high number of preset model parameter combinations were used instead of using combinations of model parameters that were obtained from genetic algorithms. Furthermore, data analysis by using full quadratic models including interaction effects did not adequately describe ADG and ADFI (data not shown). We concluded that the obtained models for ADG and ADFI from all evaluations were unsuitable, and these traits are not further described herein.

### 3.5. Gain:Feed Ratio

**Figure 2:**
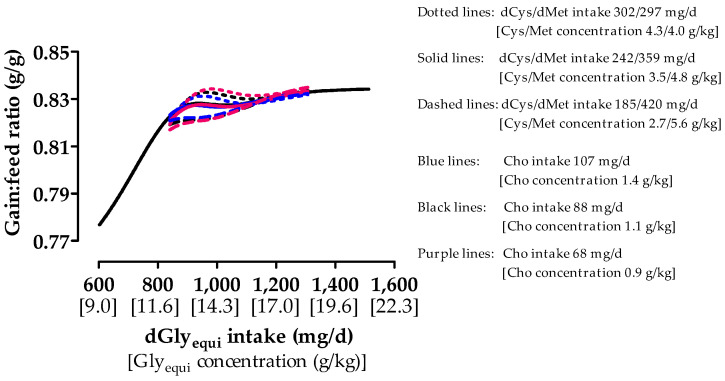
Effect of digestible glycine equivalent (dGly_equi_) intake on gain:feed ratio of broiler chickens at varying digestible cysteine (dCys) and choline (Cho) intake from days 7 to 21. dCys and Cho intake levels correspond to level −1, 0, and +1 based on the fractional central composite design.

Values of G:F from days 7 to 21 increased with dGly_equi_ intake, particularly up to a dGly_equi_ intake of 900 mg/day (Figure 2). Overall, G:F increased with higher dCys intake. The highest difference in G:F between high and low dCys intake was 0.011 g/g at a dGly_equi_ intake of 956 mg/day. The G:F was approximately 0.83 g/g with different dCys intakes and at a dGly_equi_ intake above 1170 mg/day. An effect of Cho intake on G:F was barely pronounced compared with that of dGly_equi_ and dCys intake. G:F slightly increased with low Cho and high dCys intake and the highest difference between low and high Cho intake was 0.004 g/g at 1034 mg of dGly_equi_ intake/day.

### 3.6. Nitrogen-Utilization Efficiency

**Figure 3:**
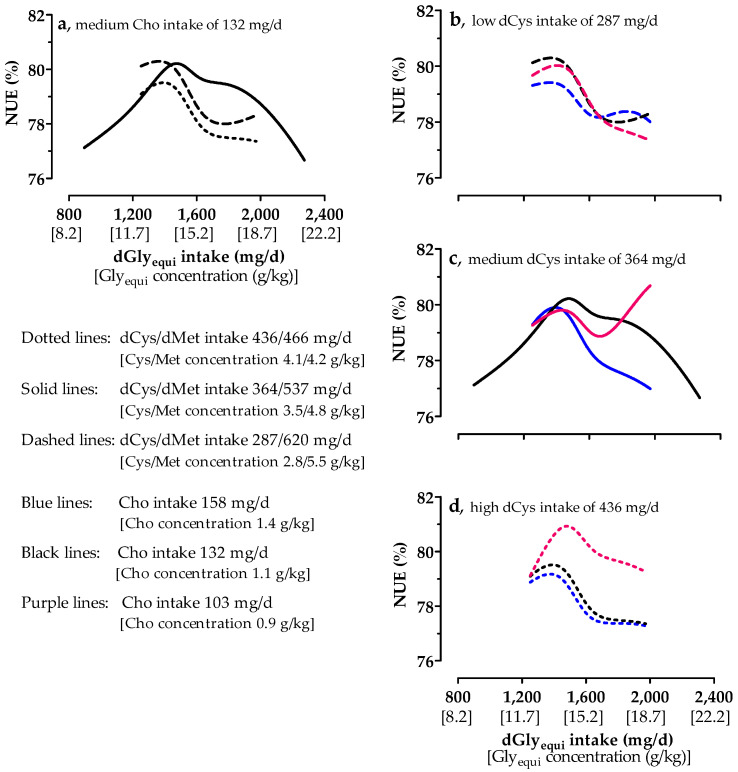
Effect of digestible glycine equivalent (dGly_equi_) intake on nitrogen−utilization efficiency (NUE) of broiler chickens at varying digestible cysteine (dCys) and medium choline (Cho) intake from days 18 to 21 (**a**), and the effect of dGly_equi_ intake on the NUE of broiler chickens at varying Cho and low, medium, and high dCys intake from days 18 to 21 (**b**–**d**). dCys and Cho intake levels correspond to level −1, 0, and +1 based on the fractional central composite design.

Values of NUE varied between 76.7% and 80.2% for medium Cho intake (Figure 3a). The NUE increased with low, medium, and high dCys intake up to a dGly_equi_ intake threshold of 1364, 1474 and 1392 mg/day, respectively, and decreased at higher intake levels of dGly_equi_. The NUE was the highest at medium dCys intake; it was higher at low dCys intake than at high dCys intake. Differences in NUE with different Cho intakes and with low dCys intake were small (Figure 3b). At medium dCys intake (Figure 3c), the influence of high and low Cho intake on NUE up to a dGly_equi_ intake of approximately 1650 mg/d was low. At high Gly_equi_ intake, NUE increased and decreased with low and high Cho intake, respectively. With high dCys intake (Figure 3d), the response to dGly_equi_ was similar with different Cho intake levels, with higher NUE at the low Cho intake compared to medium and high Cho intake.

### 3.7. Nitrogen Excretion

**Figure 4:**
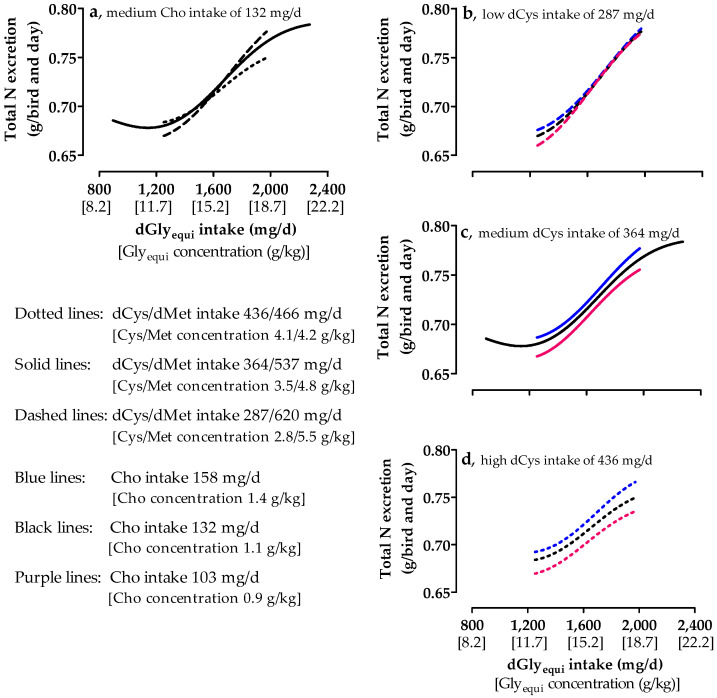
Effect of digestible glycine equivalent (dGly_equi_) intake on total nitrogen (N) excretion of broiler chickens at varying digestible cysteine (dCys) and medium choline (Cho) intake from days 18 to 21 (**a**), and effect of dGly_equi_ intake on total N excretion of broiler chickens at varying Cho and low, medium, and high dCys intake from days 18 to 21 (**b**–**d**). dCys and Cho intake levels correspond to level −1, 0, and +1 based on the fractional central composite design.

**Figure 5:**
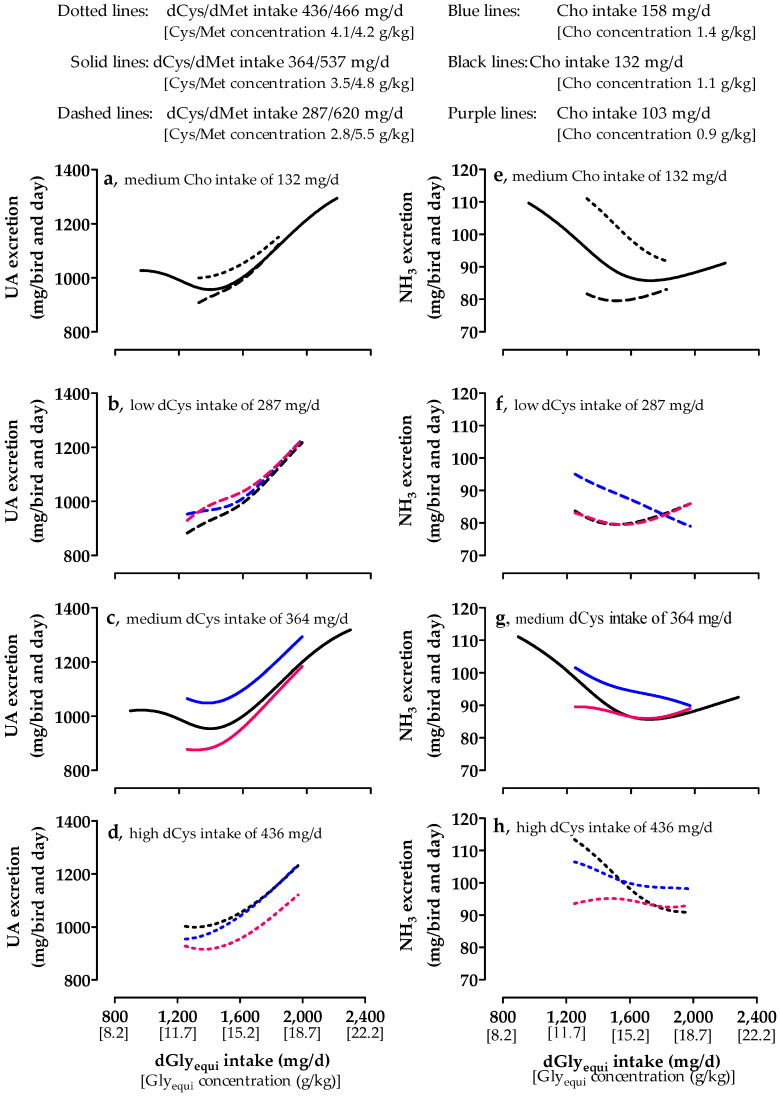
Effect of digestible glycine equivalent (dGly_equi_) intake on uric acid (UA) (**a**) and ammonia (NH_3_) (**e**) excretion of broiler chickens at varying digestible cysteine (dCys) and medium choline (Cho) intake from days 18 to 21, and the effect of dGly_equi_ intake on UA (**b**–**d**) and NH_3_ (**f**–**h**) excretion of broiler chickens at varying Cho and low, medium, and high dCys intake from days 18 and 21. dCys and Cho intake levels correspond to level −1, 0, and +1 based on the fractional central composite design.

**Figure 6:**
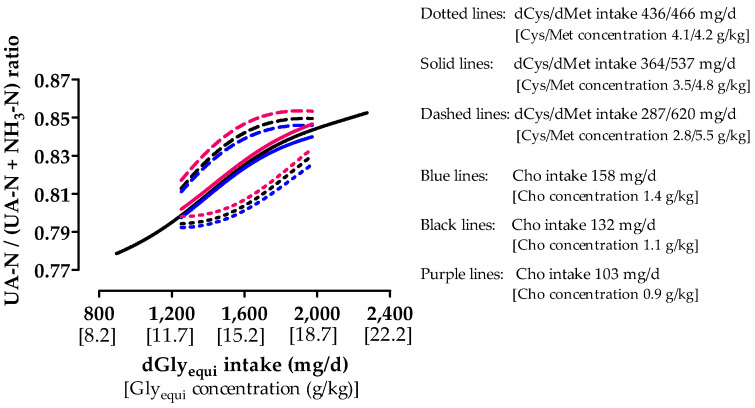
Effect of digestible glycine equivalent (dGly_equi_) intake on uric acid−nitrogen/(uric acid−nitrogen + ammonia−nitrogen) ratio ((UA-N/(UA-N + NH_3_-N) ratio) in the excreta of broiler chickens at varying digestible cysteine (dCys) and choline (Cho) intake from days 18 to 21. dCys and Cho intake levels correspond to level −1, 0, and +1 based on the fractional central composite design.

Total N and UA excretion increased with medium Cho intake when dGly_equi_ intake was higher than 1250 and 1410 mg/day, respectively (Figure 4a and Figure 5a). Lower dGly_equi_ intake did not influence total N and UA excretion. NH_3_ excretion decreased up to a dGly_equi_ intake of approximately 1700 mg/day and slightly increased at higher intake levels (Figure 5e). NH_3_ and UA excretion were the highest with high dCys intake. The intake of dCys hardly influenced total N excretion. Almost no differences in total N and UA excretion were found among different Cho intakes and with low dCys intake (Figure 4b and Figure 5b). With medium and high dCys intake, total N and UA excretion were the lowest with low Cho intake (Figure 4c,d and Figure 5c,d). NH_3_ excretion was almost unaffected by low and medium Cho intake at low and medium dCys intake (Figure 5f,g). At high dCys intake, NH_3_ excretion with medium Cho intake was the highest up to a dGly_equi_ intake of 1545 mg/day and decreased with increasing dGly_equi_ intake to the level that was excreted with low Cho intake (Figure 5h). Increasing dGly_equi_ intake and decreasing dCys intake increased the UA-N/(UA-N + NH_3_-N) ratio (Figure 6). The UA-N/(UA-N + NH_3_-N) ratio was almost unaffected by Cho intake and was slightly higher with low Cho intake.

### 3.8. Plasma Metabolites

**Figure 7:**
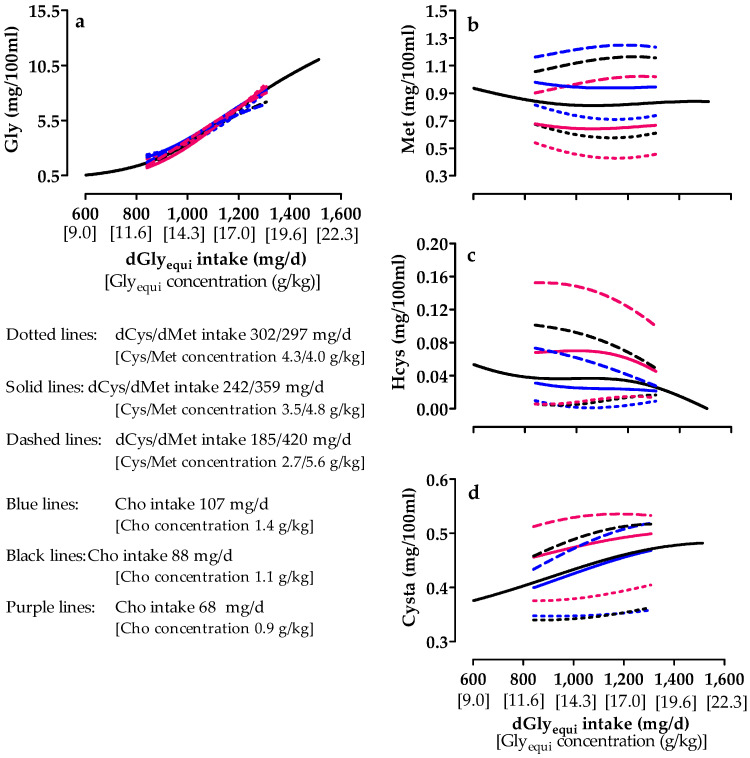
Effect of digestible glycine equivalent (dGly_equi_) intake with varying digestible cysteine (dCys) and choline (Cho) intake from days 7 to 21 on plasma glycine (Gly) (**a**), methionine (Met) (**b**), homocystine (Hcys) (**c**), and cystathionine (Cysta) (**d**) concentrations of broiler chickens on day 22. dCys and Cho intake levels correspond to level −1, 0, and +1 based on the fractional central composite design.

Increased dGly_equi_ intake increased plasma Gly concentrations and had nearly no effect on plasma Met concentrations (Figure 7a,b). Plasma Hcys concentrations decreased and plasma Cysta concentrations increased at low and medium dCys intake when dGly_equi_ intake increased (Figure 7c,d). Concentrations of these compounds were almost unaffected at high dCys intake. Plasma Met, Hcys, and Cysta concentrations decreased with increasing dCys intake. Plasma Gly concentrations were almost unaffected by dCys intake. Cho intake had nearly no effect on plasma Gly concentrations (Figure 7a). Higher Cho intake increased plasma Met concentrations irrespective of dCys intake (Figure 7b) and decreased plasma Hcys concentrations at low and medium dCys intake (Figure 7c). Plasma Hcys concentrations were almost unaffected by Cho intake at high dCys intake. The lowest Cho intake resulted in the highest plasma Cysta concentrations, and the difference in this concentration was hardly pronounced between medium and high Cho intake (Figure 7d).

## 4. Discussion

The objective of the present study was to investigate the interactive effects among dietary Gly_equi_, Cys, and Cho on growth. The interactive effects among these compounds was noted on G:F; however, they were small, although the metabolism of these compounds was influenced, as shown from the plasma metabolite concentrations. More pronounced interactive effects between Gly_equi_ and Cys [6] and between Gly_equi_ and Cho [10] on growth have been reported previously. Siegert and Rodehutscord [4] suggested that the extent of the interactive effects between Gly_equi_ and Cys, or Gly_equi_ and Cho on growth may be affected by varying NUE and urinary N excretion because the formation of UA requires Gly. The outcomes of the present study support this suggestion.

### 4.1. Effects of Gly_equi_

G:F was predominately influenced by the dGly_equi_ intake as indicated by the global sensitivity analysis. The increased NUE up to a dGly_equi_ intake of approximately 1470 mg/day with medium dCys and Cho intake showed that the more of the ingested N was accreted the less limiting Gly_equi_ intake was. This likely led to a higher G:F. NUE decreased with dGly_equi_ intake above 1470 mg/day because the additional Gly_equi_ could not be used by the animals, and the N contained in the surplus Gly_equi_ had to be excreted. Domestic fowl predominately excretes N in the urine as UA, with urinary N consisting of 72%–91% of UA−N plus NH_3_−N [23]. One molecule of Gly is required for the formation of one molecule of UA [11]. A surplus dGly_equi_ intake above 1470 mg/day was mostly excreted as UA because UA excretion and the UA-N/(UA-N + NH_3_-N) ratio increased with increasing dGly_equi_ intake. Therefore, dietary Gly_equi_ fed in excess was disadvantageous when the aim was to increase NUE.

### 4.2. Consequences of High Nitrogen-Utilization Efficiency

Varying NUE is a considerable factor that influences the effects of dietary Gly_equi_, Cys, and Cho. The observed NUE of 77% to 80% was very high compared to that found in the literature, where NUE ranged between 50% and 65% for diets containing 240 and 170 g CP/kg [24,25,26]. A high NUE indicates low catabolism of AA, meaning that digestible AA concentrations in the diets were close to the requirement of the animals. The Gly_equi_ requirement of broiler chickens decreases when NUE increases because a lower proportion of ingested N has to be excreted via the urine as UA, for which Gly is dissipated [4]. Thus, the high NUE found in the present study contributed to a low Gly_equi_ requirement of the birds. A value of 95% of maximum response, often defined as the recommended concentration of a nutrient [10,27], at a medium dCys and Cho intake was obtained at a dGly_equi_ intake of 684 mg/day (equivalent to 10 g Gly_equi_/kg). This is at the lower range of the dietary Gly_equi_ values required for broiler chickens during the first 3 weeks of age to achieve 95% of the maximum G:F that has been reported to vary between 10 and 16 g Gly_equi_/kg in previous studies [4]. Additional Gly_equi_ was possibly available when dCys (because of less Ser required to form Cys from Met) and Cho (because it is an endogenous precursor of Gly) intake increased. However, the additional Gly_equi_ obtained by these processes could not be used by the animals for G:F because the birds were abundantly supplied with Gly_equi_ in the investigated range of dGly_equi_ where the interactions with dCys and Cho could be determined (levels −1 to +1 based on the study design corresponding to 840 and 1310 mg dGly_equi_ intake/day, respectively). This could explain the small effects of varying dCys and Cho intake on G:F found in the present study. More pronounced interactive effects of dGly_equi_, dCys, and Cho intake on G:F would likely have occurred at a dGly_equi_ intake below 840 mg/d. The variation in NUE was small, which contributed to a similar Gly_equi_ requirement for UA excretion among treatments. Taken together, the high level and low variation in NUE resulted in a low Gly_equi_ requirement, which explains the small pronounced effects of dietary Cys and Cho on the response to dietary Gly_equi_ on growth.

This contradicts the findings by Siegert et al. [6], who found that the growth response to dietary Gly_equi_ was higher as dietary Cys concentrations increased. In the studies included in their meta−analysis, dietary Cys was positively correlated with dietary CP, which varied between 160 and 240 g/kg. NUE was not determined in the studies included in their meta−analysis; however, the varying dietary CP most likely influenced NUE. In contrast to the meta−analysis by Siegert et al. [6], the variation of dietary CP and NUE in the present study was low and CP and Cys concentrations in the diets were not correlated (*P* = 0.691). Therefore, the effects of different Cys concentrations in the present study were almost unaffected by the effects of different dietary CP on NUE and resulting implications on interactive effects of Gly_equi_ and Cys on G:F. The Cys effect found in the previous meta−analysis could have been an artifact caused by a concurrently variable CP supply.

### 4.3. Effects of Cysteine Formation from Methionine

Different dCys intake influenced Met conversion to Cys. This probably contributed to the overall highest G:F at high dCys intake and to higher G:F response with dGly_equi_ intake at low dCys intake compared to that at high dCys intake. These findings are consistent with the results of Siegert et al. [6] and Powell et al. [28]. The formation of Cys from Met produces Hcy and Cysta as intermediates [5]. The plasma concentrations of Hcys and Cysta varied depending on dGly_equi_ and dCys intake in the present study. The decreasing plasma Hcys and Cysta concentrations indicated that lower proportions of Met were converted to Cys with higher dCys intake. One molecule of Ser is required when Hcy is converted to Cysta [5,29], and one molecule of ammonia is produced when Cysta is metabolized to Cys [29]. Ammonia can be converted to UA during the dissipation of Gly_equi_ or it can be used to produce nonessential AA [4,30,31]. Therefore, every molecule of Met that is not metabolized to Cys spares one or two Gly_equi_ molecules. Thus, Gly_equi_ is more available for other metabolic processes such as N accretion or UA formation and could explain the slightly higher G:F and UA excretion at higher dCys intake. Conversely, more Gly_equi_ was required for Cys formation from Met to meet bird Cys requirement at lower dCys intake, which would explain the higher G:F response to Gly_equi_ supplementation at lower dCys intake than that at higher dCys intake.

### 4.4. Effects of Choline

A low conversion of Cho to Gly might be an explanation for the smaller extent of interactive effects of Gly_equi_ and Cho compared to those of Gly_equi_ and Cys on G:F. Increasing plasma concentrations of Met together with decreasing plasma concentrations of Hcys and Cysta with increasing Cho intake indicated that a higher proportion of Hcy was remethylated to Met. Remethylation of Hcy to Met can occur via the BHMT and MS pathways [29]. Via BHMT, Cho is metabolized to betaine and then to dimethylglycine when one methyl group of betaine is delivered to Hcy. Subsequently, dimethylglycine is converted to Gly. Results of studies describing whether BHMT or MS prevail upon Cho supplementation are not uniform. It was reported that Cho supplementation decreased the proportion of Hcy remethylated via BHMT [32,33] and downregulated BHMT activity [34] in broiler chickens. In this case, Cho would deliver methyl groups for the remethylation of Hcy via MS [32,33] without Gly being produced. In other studies, Cho supplementation increased BHMT activity in broiler chickens [35], pigs [36], and rats [37]. A higher activity of BHMT led to an increased formation of Gly. The reasons for these inconsistent effects of Cho supplementation on BHMT and MS activities are unclear. Possibly, the effects of Cho supplementation on BHMT and MS activities diverged among studies and, therefore, contributed to a different extent of interactive effects between dietary Cho and Gly_equi_ on growth between the results by Siegert et al. [10] and the present study.

### 4.5. Effect of Digestible Cysteine Intake on Acid Excretion

Higher dCys intake led to higher acid excretion. Increased acid excretion was indicated by the UA-N/(UA-N + NH_3_-N) ratio that increased with increasing dCys intake. The increasing UA-N/(UA-N + NH_3_-N) ratio was not caused by Gly_equi_ deficiency limiting UA formation as described previously [14,19] because Gly_equi_ was adequately supplied. Dietary Cys increased by adding Cys as free l-Cys·HCl·H_2_O, inducing an acid load in the birds owing to the contained hydrochloride [38]. Therefore, it was more likely that higher NH_3_ excretion was an adaption to the excretion of acid loads to maintain the acid−base balance [39]. Consequences of an altered acid−base balance have the potential to decrease the growth of broiler chickens [40,41]. For the present study, however, the mechanisms of the animals to maintain an acid−base balance were not challenged to an extent that decreased their growth because high dCys intake led to an overall high G:F.

### 4.6. Neural Network and Genetic Algorithm

Neural networks can be utilized to model, analyze, and optimize the response of broiler chickens to dietary nutrient concentrations or intake [10,42]. The optimal number of hidden neurons and their respective coefficients must be determined for maximized prediction accuracy. In previous studies, neural networks were often designed using a time-consuming iterative trial and error approach that requires much experience in developing neural networks by the operator. Using genetic algorithms and neural networks together can overcome these disadvantage [43,44,45]. The results of the present study indicate that using genetic algorithms for training neural networks decreases operational effort, making the architecture of neural networks less dependent of the operator. This might improve the applicability of neural networks in animal nutrition science.

## 5. Conclusions

The interactive effects of dietary Gly_equi_, Cys, and Cho on performance were slightly pronounced, possibly because NUE was high and barely influenced by the variation of these nutrients. The interactive effects between Gly_equi_ and Cho on growth were smaller than those between Gly_equi_ and Cys. Plasma metabolites indicated that the metabolism of Gly_equi_, Cys, and Cho was influenced by varying concentrations of these nutrients; however, the consequences for growth were small.

## Figures and Tables

**Table 1:** Composition of the experimental diets arranged according to the fractional central composite design (g/kg).

	Growth Trial															Digestibility Trial
Treatment	A	B	C	D	E	F	G	H	I	J	K	L	M	N	O	P
	Non-varying ingredients															
Corn	794															
Casein	84															
Soybean oil	30															
l-Arginine	7.2															
l-Aspartic acid	6.5															
l-Lysine·HCl	4.8															
l-Valine	3.8															
l-Threonine	2.6															
l-Alanine	2.2															
l-Isoleucine	2.2															
l-Glutamic acid	1.8															
l-Phenylalanine	1.1															
l-Tryptophan	0.4															
l-Tyrosine	0.2															
Limestone	15.3															
Monocalcium phosphate	15.0															
Sodium bicarbonate	3.3															
Vitamin premix ^1^	2.0															
Sodium chloride	1.0															
Trace element premix ^2^	0.5															
	Varying ingredients															
dl-Methionine	1.7	2.5	2.5	0.8	0.8	3.4	1.7	1.7	1.7	-	2.5	2.5	0.8	0.8	1.7	-
l-Cysteine·HCl·H_2_O	3.2	1.7	1.7	4.6	4.6	0.3	3.2	3.2	3.2	6.0	1.7	1.7	4.6	4.6	3.2	0.3
Glycine	0.2	3.4	3.4	3.4	3.4	6.6	6.6	6.6	6.6	6.6	9.7	9.7	9.7	9.7	12.9	0.2
Choline chloride ^3^	2.6	1.6	3.7	1.6	3.7	2.6	0.5	2.6	4.7	2.6	1.6	3.7	1.6	3.7	2.6	2.6
Cornstarch	14.4	12.9	10.8	11.7	9.6	9.2	10.1	8.0	5.9	6.9	6.6	4.5	5.4	3.3	1.7	14.0
TiO_2_	-	-	-	-	-	-	-	-	-	-	-	-	-	-	-	5.0

^1^ Vitamin premix (Miavit GmbH, Essen, Germany) provided per kg of diet: 10,000 IU vitamin A as retinyl acetate (3a672a); 3000 IU vitamin D3 as cholecalciferol (E671); 30 IU vitamin E as all rac-α-tocopherol (3a700); 2.4 mg vitamin K3 as menadione (3a711); 100 µg biotin (3a880); 1 mg folic acid (3a316); 3 mg thiamine (3a821); 6 mg riboflavin; 6 mg pyridoxine (3a831); 30 µg vitamin B12; 50 mg nicotinamide (3a315); 14 mg calcium D-pantothenate (3a841). ^2^ Trace element premix (Gelamin Gesellschaft für Tierernährung mbH, Memmingen, Germany) provided per kg of diet: 25 mg calcium from calcium carbonate; 80 mg manganese from manganese-(II)-oxide; 60 mg zinc from zinc-sulfate; 25 mg iron from ferrous-(II)-sulfate monohydrate; 7.5 mg copper from cupric-(II)-sulfate pentahydrate; 0.6 mg iodine from calcium iodate; 0.2 mg selenium from sodium selenite; 15 mg sepiolite. ^3^ 50% Choline chloride, 373 g/kg choline concentration.

**Table 2:** Combinations of different nutrients and calculated and analyzed nutrient concentrations (g/kg) of glycine equivalent (Gly_equi_), cysteine (Cys), and choline (Cho) in the experimental diets.

Treatment	Level Combinations ^1^			Calculated Nutrient Concentrations			Analyzed Nutrient Concentrations		
	Gly_equi_	Cys	Cho	Gly_equi_	Cys	Cho ^2^	Gly_equi_	Cys	Cho ^3^
Growth trial									
A	−2	0	0	9	3.5	1.1	9.1	3.5	n. a.
B	−1	−1	−1	12	2.75	0.8	12.2	2.6	n. a.
C	−1	−1	1	12	2.75	1.4	12.0	2.6	1.4
D	−1	1	−1	12	4.25	0.8	12.0	4.4	1.0
E	−1	1	1	12	4.25	1.4	12.2	4.3	1.6
F	0	−2	0	15	2.0	1.1	14.9	1.8	n. a.
G	0	0	−2	15	3.5	0.5	15.1	3.4	0.8
H (central diet)	0	0	0	15	3.5	1.1	15.1	3.4	1.3
I	0	0	2	15	3.5	1.7	15.1	3.5	2.1
J	0	2	0	15	5.0	1.1	15.0	5.1	n. a.
K	1	−1	−1	18	2.75	0.8	18.1	2.7	0.9
L	1	−1	1	18	2.75	1.4	18.2	2.7	1.5
M	1	1	−1	18	4.25	0.8	18.4	4.4	n. a.
N	1	1	1	18	4.25	1.4	18.6	4.4	1.4
O	2	0	0	21	3.5	1.1	21.3	3.6	1.3
Digestibility trial									
P	−2	−2	0	9	2.0	1.1	8.9	1.8	n. a.

^1^ According to the fractional central composite design for treatments A–O; ^2^ Calculated concentrations were used for further calculations; ^3^ n. a. = not analyzed.

**Table 3:** Information and criteria of the fit of the neural network models for the prediction of gain:feed ratio of broiler chickens from days 7 to 21, and excreta characteristics of broiler chickens from days 18 to 21 based on the dietary intake of digestible glycine equivalent, digestible cysteine, and total choline.

Item	G:F		NUE		Daily NH_3_ Excretion		Daily UA Excretion		UA-N/(UA-N + NH_3_-N) Ratio		Daily N Excretion	
	Training Set	Testing Set	Training Set	Testing Set	Training Set	Testing Set	Training Set	Testing Set	Training Set	Testing Set	Training Set	Testing Set
Type of network	Fully connected feed forward network											
Training algorithm	Evolutionary training using Genetic Algorithms											
Network structure selection	Genetic algorithms to identify neural network topology											
No. of observations in training and testing set	73	32	71	30	70	31	70	31	71	30	70	31
Optimal number of hidden neurons identified by genetic algorithms	4		5		4		8		5		4	
Type of activation function in hidden neuron	Hyperbolic tangent		Hyperbolic tangent		Hyperbolic tangent		Hyperbolic tangent		Hyperbolic tangent		Hyperbolic tangent	
Type of basing function in output neuron	Linear		Linear		Linear		Linear		Linear		Linear	
R^2^	0.75	0.90	0.60	0.66	0.83	0.90	0.83	0.84	0.85	0.82	0.54	0.63
R^2^ of the whole data set	0.81		0.61		0.86		0.83		0.84		0.56	
Root MSE	0.007 g/g	0.006 g/g	0.92%	0.93%	4.48 mg/bird	4.55 mg/bird	59.0 mg/bird	54.0 mg/bird	0.01	0.01	0.04 g/bird	0.03 g/bird
Root MSE of the whole data set	0.007 g/g		0.92%		4.75 mg/bird		57.5 mg/bird		0.01		0.03 g/bird	

G:F, gain:feed ratio; NUE, nitrogen−utilization efficiency; NH_3_, ammonia; UA, uric acid; UA-N/(UA-N + NH_3_-N) ratio, uric acid−nitrogen/(uric acid-nitrogen + ammonia-nitrogen) ratio; N, nitrogen.

**Table 4:** Information and criteria of the fit of the neural network models for the prediction of some plasma amino acids and biogenic amines in broiler chickens on day 22 based on a dietary intake of digestible glycine equivalent, digestible cysteine, and total choline.

	Gly		Met		Hcys ^1^		Cysta ^2^	
Item	Training Set	Testing Set	Training Set	Testing Set	Training Set	Testing Set	Training Set	Testing Set
n	Fully connected feed forward network							
Training algorithm	Evolutionary training using Genetic Algorithms							
Network structure selection	Genetic algorithms to identify neural network topology							
No. of observations in training and testing set	72	31	72	31	72	31	64	28
Optimal number of hidden neurons identified by genetic algorithms	7		7		7		6	
Type of activation function in hidden neuron	Hyperbolic tangent		Hyperbolic tangent		Hyperbolic tangent		Hyperbolic tangent	
Type of basing function in output neuron	Linear		Linear		Linear		Linear	
R^2^	0.81	0.77	0.67	0.67	0.50	0.65	0.47	0.63
R^2^ of the whole data set	0.80		0.67		0.55		0.51	
Root MSE (mg/100 mL)	1.45	1.69	0.27	0.24	0.05	0.07	0.09	0.07
Root MSE of the whole data set (mg/100 mL)	1.53		0.26		0.06		0.09	

^1^ 55 observations set to 0 because no concentrations were detectable, and the lowest quantified concentration of 0.05 mg/100 mL was close to 0; ^2^ 11 observations were not used for data evaluation because no concentrations were quantified, and the lowest detected concentration (0.16 mg/100 mL) was not close to 0; Gly, glycine; Met, methionine; Hcys, homocystine; Cysta, cystathionine.

**Table 5:** Prececal digestibility (%, arithmetic mean ± SD) of crude protein and amino acids in the digestibility trial diet (n = 5).

Item	Prececal Digestibility
Crude protein	91 ± 0.8
Lysine	95 ± 0.9
Methionine	95 ± 0.9
Cysteine	81 ± 1.2
Threonine	89 ± 0.9
Tryptophan	90 ± 1.8
Arginine	96 ± 0.6
Valine	93 ± 0.6
Isoleucine	92 ± 0.8
Leucine	94 ± 0.6
Histidine	91 ± 0.8
Phenylalanine	96 ± 0.7
Glycine	84 ± 1.5
Serine	84 ± 1.2
Alanine	93 ± 0.8
Aspartic acid/asparagine	93 ± 0.8
Glutamic acid/glutamine	92 ± 0.7
Proline	92 ± 0.4

**Table 6:** Variable total effect in the neural network models for the prediction of gain:feed ratio, excreta characteristics, and plasma metabolites.

Item	dGly_equi_ Intake	dCys Intake	Cho Intake
G:F			
Sensitivity	0.99	0.04	0.02
Rank	1	2	3
NUE			
Sensitivity	0.78	0.55	0.35
Rank	1	2	3
Total N excretion			
Sensitivity	0.93	0.08	0.09
Rank	1	3	2
UA excretion			
Sensitivity	0.89	0.19	0.11
Rank	1	2	3
NH_3_ excretion			
Sensitivity	0.32	0.72	0.38
Rank	3	1	2
UA-N/(UA-N + NH_3_-N) ratio			
Sensitivity	0.63	0.54	0.06
Rank	1	2	3
Plasma Gly concentration			
Sensitivity	0.97	0.06	0.04
Rank	1	2	3
Plasma Met concentration			
Sensitivity	0.05	0.81	0.20
Rank	3	1	2
Plasma Hcys concentration			
Sensitivity	0.15	0.70	0.20
Rank	3	1	2
Plasma Cysta concentration			
Sensitivity	0.10	0.87	0.13
Rank	3	1	2

dGly_equi_, digestible glycine equivalent; dCys, digestible cysteine; Cho, choline; G:F, gain:feed ratio; NUE, nitrogen−utilization efficiency; N, nitrogen; UA, uric acid; NH_3_, ammonia; UA-N/(UA-N + NH_3_-N) ratio, uric acid-nitrogen/(uric acid-nitrogen + ammonia-nitrogen) ratio; Gly, glycine; Met, methionine; Hcy, homocystine; Cysta, cystathionine.

## Supplementary Materials

The following are available online at https://www.mdpi.com/2076-2615/10/8/1392/s1, Figure S1: Effect of digestible glycine equivalent (dGly_equi_) intake on average daily gain (ADG) of broiler chickens at varying digestible cysteine (dCys) and medium choline (Cho) intake from days 7 to 21 (**a**). The effect of dGly_equi_ intake on ADG of broiler chickens at varying Cho and low, medium, and high dCys intake from days 7 to 21 (**b–d**). dCys and Cho intake levels correspond to level −1, 0, and +1 based on the fractional composite design. Figure S2: Effect of digestible glycine equivalent (dGly_equi_) intake on average daily feed intake (ADFI) of broiler chickens at varying digestible cysteine (dCys) and medium choline (Cho) intake from days 7 to 21 (**a**) and effects of dGly_equi_ intake on ADFI of broiler chickens at varying Cho and low, medium, and high dCys intake from days 7 to 21 (**b–d**). dCys and Cho intake levels correspond to level −1, 0, and +1 based on the fractional composite design. Table S1: Analyzed concentrations of nutrients other than glycine equivalent, cysteine, and choline of the experimental diets (g/kg on a 88% dry matter basis unless otherwise stated). Table S2: Neural network model equations for gain:feed ratio, excreta characteristics, and blood metabolites. Table S3: Description of neural network models for the prediction of traits that were not further described because R^2^ was ≤ 0.50 or because model results were difficult to interpret biologically.
