# Supplementary material for: Interactive Effects of Glycine Equivalent, Cysteine, and Choline on Growth Performance, Nitrogen Excretion Characteristics, and Plasma Metabolites of Broiler Chickens Using Neural Networks Optimized with Genetic Algorithms
Source: Animals (Basel). 2020 Aug 11;10(8):1392. doi: 10.3390/ani10081392 (PMC7459920; doi:10.3390/ani10081392)
Supplement: Supplementary file 1 [file animals-10-01392-s001.pdf]

Interactive effects of glycine equivalent, cysteine, and choline on growth performance, nitrogen excretion characteristics, and plasma metabolites of broiler chickens using neural networks optimized with genetic algorithms

Supplementary data

Table of contents

Table S1. Analyzed nutrient concentrations other than glycine equivalent, cysteine, and choline of the experimental diets. 2

Table S2. Neural network model equations for gain:feed ratio, excreta characteristics, and blood metabolites. 3

Table S3. Description of neural network models for the prediction of traits that were not described in the main manuscript. 5

Figure S1. Average daily gain. 6

Figure S2. Average daily feed intake. 7

References 7

Table S1. Analyzed concentrations of nutrients other than glycine equivalent, cysteine, and choline of the experimental diets (g/kg on a 88% dry matter basis unless otherwise stated).

	Growth trial															Digestibility trial
Treatment	A	B	C	D	E	F	G	H	I	J	K	L	M	N	O	P
Dry matter (g/kg)	911	913	911	913	913	912	912	910	911	911	912	914	914	913	914	909
Crude fat[1]	n. a.	n. a.	n. a.	n. a.	n. a.	n. a.	n. a.	67.6	n. a.	n. a.	n. a.	n. a.	n. a.	n. a.	n. a.	n. a.
Crude fiber[1]	n. a.	n. a.	n. a.	n. a.	n. a.	n. a.	n. a.	15.7	n. a.	n. a.	n. a.	n. a.	n. a.	n. a.	n. a.	n. a.
Crude ash[1]	n. a.	n. a.	n. a.	n. a.	n. a.	n. a.	n. a.	39.2	n. a.	n. a.	n. a.	n. a.	n. a.	n. a.	n. a.	n. a.
Starch[1]	n. a.	n. a.	n. a.	n. a.	n. a.	n. a.	n. a.	531	n. a.	n. a.	n. a.	n. a.	n. a.	n. a.	n. a.	n. a.
AME_N (MJ/kg)[2,3]	n. c.	n. c.	n. c.	n. c.	n. c.	n. c.	n. c.	14.0	n. c.	n. c.	n. c.	n. c.	n. c.	n. c.	n. c.	n. c.
Crude protein	172	175	174	175	175	178	178	176	178	179	182	182	182	183	185	165
Lysine	11.9	11.9	11.6	11.9	11.8	11.5	11.7	11.5	11.7	11.5	11.9	11.7	11.9	11.9	11.7	11.3
Methionine	5.0	5.8	5.6	4.2	4.2	6.5	4.9	4.9	4.9	3.3	5.8	5.7	4.2	4.2	5.0	3.3
Methionine+Cysteine	8.5	8.4	8.2	8.5	8.5	8.3	8.3	8.3	8.4	8.4	8.5	8.5	8.6	8.6	8.5	5.1
Threonine	7.8	7.8	7.7	7.7	7.8	7.7	7.7	7.7	7.8	7.7	7.8	7.8	7.9	7.9	7.8	7.5
Tryptophan	1.4	1.8	1.8	1.8	1.8	1.9	1.9	1.8	1.8	1.8	2.0	2.0	2.0	2.0	2.4	2.1
Arginine	12.5	12.5	12.2	12.6	12.4	12.1	12.3	12.2	12.4	12.2	12.5	12.4	12.5	12.5	12.4	11.9
Isoleucine	8.2	8.1	8.0	8.1	8.1	8.0	8.0	7.9	8.0	7.8	8.2	8.2	8.0	8.3	8.2	7.9
Leucine	14.2	14.2	14.1	14.1	14.3	14.2	14.1	14.0	14.2	14.0	14.4	14.2	14.2	14.3	14.3	14.0
Valine	11.5	11.4	11.3	11.3	11.4	11.3	11.4	11.2	11.4	11.1	11.4	11.4	11.5	11.5	11.5	11.0
Histidine	3.9	3.9	3.8	3.9	3.9	3.8	3.8	3.8	3.9	3.7	4.1	4.1	3.9	4.1	4.1	3.9
Phenylalanine	7.9	7.9	7.8	7.9	7.9	7.8	7.8	7.7	7.9	7.7	7.8	7.8	7.8	7.8	7.8	7.6
Proline	13.0	13.1	12.9	13.6	13.0	13.0	12.9	12.9	13.1	12.8	13.6	13.4	13.2	13.7	13.5	13.3
Alanine	8.8	8.8	8.8	8.8	8.9	8.8	8.8	8.8	8.9	8.8	8.8	8.8	8.9	8.9	8.9	8.6
Aspartic acid/asparagine	15.8	15.8	15.5	15.7	15.7	15.4	15.5	15.5	15.7	15.5	15.8	15.7	15.9	16.0	15.7	15.2
Glutamic acid/glutamine	29.5	29.7	29.3	29.0	29.8	29.4	29.2	29.0	29.5	29.1	29.4	29.1	29.7	29.7	29.3	28.5
Glycine	4.0	7.0	6.9	7.0	7.0	9.8	10.0	10.0	10.0	9.9	13.0	13.1	13.2	13.4	16.2	3.9
Serine	7.2	7.3	7.1	7.1	7.3	7.2	7.1	7.2	7.2	7.2	7.2	7.1	7.3	7.3	7.1	7.0
Methionine/(Methionine+Cysteine) ratio	0.59	0.69	0.69	0.49	0.49	0.78	0.59	0.58	0.59	0.39	0.69	0.68	0.49	0.49	0.58	0.65

[1]n. a. = not analyzed.

[2]Apparent metabolizable energy (nitrogen corrected), calculated according to the estimation equation of the WPSA [1]. Analyzed nutrient concentrations and table values for sugar in corn and casein [2] were used.

[3]n. c. = not calculated.

Table S2. Neural network model equations for gain:feed ratio, excreta characteristics, and blood metabolites.

Traits	Equations[1–5]
Gain:feed ratio	
Hidden neuron 1 (HN1)	TanH(0.5 × (0.00745711012749822 × $dGly_{equi}$ + 0.00698451302839905 × Cho – 0.00265372829160152 × dCys – 5.96002791011724))
Hidden neuron 2 (HN2)	TanH(0.5 × (0.00596337064949425 × $dGly_{equi}$ – 0.0698739788974951 × Cho – 0.0402240341970197 × dCys + 8.69003674390456))
Hidden neuron 3 (HN3)	TanH(0.5 × (0.0138811579639639 × $dGly_{equi}$ + 0.00960315986792434 × Cho – 0.0118314249050328 × dCys – 10.912831676772))
Hidden neuron 4 (HN4)	TanH(-0.5 × (0.000424187683443493 × $dGly_{equi}$ + 0.0218352158546493 × Cho + 0.00749272187491747 × dCys – 3.64290746493713))
Output neuron[6]	0.0763486603283619 × HN1 – 0.00380518773896112 × HN2 – 0.0317937180645929 × HN3 – 0.0136298923246927 × HN4 + 0.789410842591448
Nitrogen-utilization efficiency	
Hidden neuron 1 (HN1)	TanH(0.5 × 0.00238809431086125 × $dGly_{equi}$ + 0.00369959486073259 × Cho – 0.00578061658686241 × dCys – 4.94794411961778))
Hidden neuron 2 (HN2)	TanH(0.5 × 0.0163938381235982 × $dGly_{equi}$ + 0.0107924760520368 × Cho + 0.00038146070118685 × dCys – 26.8918164433813))
Hidden neuron 3 (HN3)	TanH(0.5 × 0.00212482132418724 × $dGly_{equi}$ + 0.073800934463446 × Cho + 0.0297311152065488 × dCys – 23.9834372868202))
Hidden neuron 4 (HN4)	TanH(0.5 × 0.00636065423886399 × $dGly_{equi}$ + 0.0943445503091565 × Cho + 0.0497882815647489 × dCys – 39.8529588059288))
Hidden neuron 5 (HN5)	TanH(0.5 × 0.00116020846578174 × $dGly_{equi}$ + 0.00589608764376348 × Cho – 0.000933189827134606 × dCys – 1.15350111684146))
Output neuron[6]	-0.107970644682149 × HN1 – 0.0150777973420628 × HN2 – 0.0457133498344239 × HN3 + 0.0336345784606326 × HN4 + 0.115463172640225 × HN5 + 0.634732458028624
Nitrogen excretion	
Hidden neuron 1 (HN1)	TanH(0.5 × (0.000982998184595515 × $dGly_{equi}$ – 0.0168628113560583 × Cho + 0.0140056587305307 × dCys – 5.72534466376565))
Hidden neuron 2 (HN2)	TanH(0.5 × (-0.00316435200286084 × $dGly_{equi}$ – 0.0028229635168365 × Cho + 0.0122063842336211 × dCys – 1.18382950621916))
Hidden neuron 3 (HN3)	TanH(0.5 × (-0.00309788938051577 × $dGly_{equi}$ + 0.0262905279689474 × Cho + 0.00405662189811896 × dCys – 0.109618304015416))
Hidden neuron 4 (HN4)	TanH(0.5 × (0.00367127212551882 × $dGly_{equi}$ – 0.0110342414823243 × Cho + 0.000508397293516325 × dCys – 4.69089823709291))
Output neuron[6]	-0.0618729913433882 × HN1 + 0.0452287736477285 × HN2 + 0.0645760392669033 × HN3 + 0.160611942421598 × HN4 + 0.72750952309789
Uric acid excretion	
Hidden neuron 1 (HN1)	TanH(0.5 × (-0.00115725766622595 × $dGly_{equi}$ – 0.0392830222796235 × Cho – 0.0246043442559308 × dCys + 16.8995356254814))
Hidden neuron 2 (HN2)	TanH(0.5 × (-0.00332863851872401 × $dGly_{equi}$ – 0.00568348355114068 × Cho + 0.00292371086787016 × dCys + 5.92460137522459))
Hidden neuron 3 (HN3)	TanH(0.5 × (-0.00410701138428459 × $dGly_{equi}$ + 0.0357877884966724 × Cho + 0.0177396479771911 × dCys – 3.84691541001452))
Hidden neuron 4 (HN4)	TanH(0.5 × (0.00205511682804899 × $dGly_{equi}$ – 0.000568118351311531 × Cho + 0.011322098927765 × dCys – 5.72446515240262))
Hidden neuron 5 (HN5)	TanH(0.5 × (0.0011224125896147 × $dGly_{equi}$ – 0.0731670922711829 × Cho – 0.0321173449351992 × dCys + 20.036741036197))
Hidden neuron 6 (HN6)	TanH(0.5 × (0.00821205986304593 × $dGly_{equi}$ + 0.0088462201788829 × Cho + 0.0230939964593827 × dCys – 19.8019722952964))
Hidden neuron 7 (HN7)	TanH(0.5 × (0.00117032234144227 × $dGly_{equi}$ – 0.0018402289140217 × Cho – 0.0124571150175835 × dCys + 2.51533213049508))
Hidden neuron 8 (HN8)	TanH(0.5 × (-0.0002510537348747 × $dGly_{equi}$ + 0.0387244230451899 × Cho – 0.00452540740759516 × dCys – 4.60221286337894))
Output neuron[6]	193.605934420824 × HN1 – 269.766798887977 × HN2 – 127.468695734284 × HN3 + 265.938421034167 × HN4 – 265.969511267426 × HN5 – 130.772578853385 × HN6 + 12.229767773722 × HN7 – 19.2843049655749 × HN8 + 1072.97981260586
NH_3 excretion	
Hidden neuron 1 (HN1)	TanH(0.5 × (0.00118671405026021 × $dGly_{equi}$ + 0.121856456729561 × Cho + 0.00567003669843782 × dCys – 22.3537117877435))
Hidden neuron 2 (HN2)	TanH(0.5 × (0.0068858102189526 × $dGly_{equi}$ + 0.0328955561125764 × Cho – 0.0163022282676837 × dCys – 7.8923089300079))
Hidden neuron 3 (HN3)	TanH(0.5 × (-0.00379723894018782 × $dGly_{equi}$ + 0.157157898515468 × Cho + 0.0152060150732619 × dCys – 22.3203685353457))
Hidden neuron 4 (HN4)	TanH(0.5 × (0.00155726211109731 × $dGly_{equi}$ + 0.00647524059969059 × Cho + 0.0020401940335174 × dCys – 4.5236839176507))
Output neuron[6]	-18.9820572641769·× HN1 – 10.2420061382676 × HN2 + 19.8153981877952 × HN3 + 32.871286466422 × HN4 + 100.152854408833
Uric acid-nitrogen/(uric acid-nitrogen+ammonia-nitrogen) ratio	
Hidden neuron 1 (HN1)	TanH(0.5 × (0.00240101502267026 × $dGly_{equi}$ – 0.00676164953397091 × Cho + 0.01012236037695 × dCys – 6.94416622338394))
Hidden neuron 2 (HN2)	TanH(0.5 × (-0.00153705628777243 × $dGly_{equi}$ + 0.0204124271251688 × Cho – 0.00363782611157049 × dCys + 1.38354050255561))
Hidden neuron 3 (HN3)	TanH(0.5 × (-0.00020835328942386 × $dGly_{equi}$ – 0.00156908717050396 × Cho – 0.0121803246827632 × dCys + 4.71920483995858))
Hidden neuron 4 (HN4)	TanH(0.5 × (-0.00182955082807785 × $dGly_{equi}$ – 0.0000504981475518491 × Cho + 0.0166590066316234 × dCys – 2.26373756981236))
Hidden neuron 5 (HN5)	TanH(0.5 × (0.00403595898960563 × $dGly_{equi}$ + 0.00315495038620843 × Cho – 0.0164494310574208 × dCys – 0.250553977719294))
Output neuron[6]	0.071778191069152 × HN1 + 0.0455790608586901 × HN2 + 0.0978065018342792 × HN3 + 0.0397960569757376 × HN4 – 0.0426506508103158 × HN5 + 0.188523293986036

Table continued on next page

Table S2. Continuation.

Plasma glycine concentration	
Hidden neuron 1 (HN1)	TanH(0.5 × (0.000760000429386617 × $dGly_{equi}$ – 0.0100168431168882 × Cho + 0.00612558406762568 × dCys – 0.525108975849258))
Hidden neuron 2 (HN2)	TanH(0.5 × (0.0000996704329709996 × $dGly_{equi}$ + 0.0159030428954029 × Cho – 0.0205677378809105 × dCys + 4.23374396341638))
Hidden neuron 3 (HN3)	TanH(0.5 × (0.000743135986553323 × $dGly_{equi}$ – 0.0293649257778842 × Cho – 0.0239091364017607 × dCys + 10.2723248077175))
Hidden neuron 4 (HN4)	TanH(0.5 × (0.00342357184779043 × $dGly_{equi}$ – 0.0231122058959766 × Cho + 0.00629930317973919 × dCys – 4.94985725165811))
Hidden neuron 5 (HN5)	TanH(0.5 × (0.000219915696046319 × $dGly_{equi}$ + 0.0173352378918462 × Cho – 0.017889393629971 × dCys + 2.93323204229481))
Hidden neuron 6 (HN6)	TanH(0.5 × (0.0000799813916047737 × $dGly_{equi}$ – 0.0679509987660939 × Cho – 0.0326053532160734 × dCys + 13.7034555024411))
Hidden neuron 7 (HN7)	TanH(0.5 × (0.00695716417160134 × $dGly_{equi}$ + 0.00492729668663004 × Cho – 0.0184009164707665 × dCys – 3.66825370484023))
Output neuron[6]	0.872859520864669 × HN1 + 4.80640054759043 × HN2 – 1.01936082435685 × HN3 + 5.57878052352918 × HN4 – 4.38102299953961 × HN5 – 1.39255190264167 × HN6 + 3.36768297209901 × HN7 + 8.37788956515117
Plasma methionine concentration	
Hidden neuron 1 (HN1)	TanH(0.5 × (-0.00449373736622504 × $dGly_{equi}$ + 0.036849618432971 × Cho – 0.0174359178343077 × dCys + 4.87391665440295))
Hidden neuron 2 (HN2)	TanH(0.5 × (0.00137044708430015 × $dGly_{equi}$ + 0.00579062568171863 × Cho + 0.0124751949849345 × dCys – 5.05027522006035))
Hidden neuron 3 (HN3)	TanH(0.5 × (0.00277450614915356 × $dGly_{equi}$ – 0.00534904333058834 × Cho + 0.0145176517177475 × dCys – 6.07330149086107))
Hidden neuron 4 (HN4)	TanH(0.5 × (-0.00078419883260005 × $dGly_{equi}$ + 0.0278964535752436 × Cho + 0.0279575740070765 × dCys – 7.95140378229135))
Hidden neuron 5 (HN5)	TanH(0.5 × (-0.00063027106759293 × $dGly_{equi}$ + 0.00482156215949677 × Cho + 0.0266194042049553 × dCys – 6.57716755102992))
Hidden neuron 6 (HN6)	TanH(0.5 × (-0.00111394925914072 × $dGly_{equi}$ – 0.0605801303229812 × Cho + 0.00801074171641 × dCys + 4.04943981284606))
Hidden neuron 7 (HN7)	TanH(0.5 × (-0.00403071757465593 × $dGly_{equi}$ – 0.000966709670200468 × Cho + 0.0155585152091057 × dCys + 1.19317136771864))
Output neuron[6]	0.0110560118056625 × HN1 – 0.615579340849217 × HN2 – 0.388354017259169 × HN3 + 0.0318756416440476 × HN4 + 0.541551646848177 × HN5 – 0.243942886158744 × HN6 – 0.556703527685973 × HN7 + 0.971971954957111
Plasma homocystine concentration	
Hidden neuron 1 (HN1)	TanH(0.5 × (-0.00477804072311765 × $dGly_{equi}$ + 0.0148889535310467 × Cho + 0.0181523000593003 × dCys – 0.96837855729247))
Hidden neuron 2 (HN2)	TanH(0.5 × (0.000999197756528903 × $dGly_{equi}$ + 0.0527025034558708 × Cho + 0.0163050170048935 × dCys – 10.0313460802819))
Hidden neuron 3 (HN3)	TanH(0.5 × (-0.0000618002131480893 × $dGly_{equi}$ + 0.0236368254276596 × Cho + 0.00881662137837281 × dCys – 4.71368397352631))
Hidden neuron 4 (HN4)	TanH(0.5 × (0.00143739714975731 × $dGly_{equi}$ + 0.0234249871752812 × Cho + 0.0106154330857532 × dCys – 5.64919064897512))
Hidden neuron 5 (HN5)	TanH(0.5 × (-0.0050133373542686 × $dGly_{equi}$ + 0.0311152499613936 × Cho – 0.00257353029033734 × dCys + 3.51229800487801))
Hidden neuron 6 (HN6)	TanH(0.5 × (-0.00487996772770758 × $dGly_{equi}$ – 0.0331788089268689 × Cho + 0.00239268970256211 × dCys + 9.17389580364018))
Hidden neuron 7 (HN7)	TanH(0.5 × (0.00455700491517116 × $dGly_{equi}$ + 0.0160614281928823 × Cho – 0.00968843061845564 × dCys – 4.09273752085958))
Output neuron[6]	-0.0788554018649295 × HN1 + 0.129495229761163 × HN2 + 0.12788015485832 × HN3 – 0.350355530957148 × HN4 – 0.042363368196125 × HN5 + 0.0448938346810705 × HN6 – 0.0228464634372538 × HN7 + 0.144468983952688
Plasma cystathionine concentration	
Hidden neuron 1 (HN1)	TanH(0.5 × (0.0018847108303394 × $dGly_{equi}$ – 0.0167973950735392 × Cho + 0.00609149607620916 × dCys – 2.45086355248683))
Hidden neuron 2 (HN2)	TanH(0.5 × (-0.0000338194968962919 × $dGly_{equi}$ – 0.055705671068942 × Cho + 0.000465465734435653 × dCys + 3.44232933353857))
Hidden neuron 3 (HN3)	TanH(0.5 × (0.0010479345286331 × $dGly_{equi}$ + 0.00115813835757484 × Cho + 0.0336284246108703 × dCys – 8.8088124478422))
Hidden neuron 4 (HN4)	TanH(0.5 × (-0.00708022360854824 × $dGly_{equi}$ + 0.029204796344125 × Cho + 0.0018363262130015 × dCys + 5.26359754114427))
Hidden neuron 5 (HN5)	TanH(0.5 × (0.00260503147968808 × $dGly_{equi}$ – 0.0211313452914882 × Cho – 0.0100052421334673 × dCys + 1.48154695100932))
Hidden neuron 6 (HN6)	TanH(0.5 × (0.00232632663152387 × $dGly_{equi}$ + 0.0301340324533397 × Cho – 0.00414175555010358 × dCys – 4.27356927379416))
Output neuron[6]	-0.415061839233271 × HN1 + 0.162791647357952 × HN2 + 0.111828856967947 × HN3 + 0.0011353155133886 × HN4 + 0.27772695399449 × HN5 + 0.0903026821642312 × HN6 + 0.428873743184756

[1]TanH: Hyperbolic tangent.

[2]Numbers that are multiplied with $dGly_{equi}$ are the weights for variable 1 ($dGly_{equi}$) in the respective hidden neuron.

[3]Numbers that are multiplied with Cho are the weights for variable 2 (Cho) in the respective hidden neuron.

[4]Numbers that are multiplied with dCys are the weights for variable 3 (dCys) in the respective hidden neuron.

[5]Last numbers in the equation of each hidden neuron represent the bias value.

[6]Linear combination of the equations that predict the respective trait. There are 4–8 weights (depending on the number of hidden neurons) multiplied with the equation of the respective hidden neuron and a bias value at the end.

$dGly_{equi}$, digestible glycine equivalent; dCys, digestible cysteine; Cho, choline.

Table S3. Description of neural network models for the prediction of traits that were not further described because R^2 was ≤ 0.50 or because model results were difficult to interpret biologically.

Trait	Observed range	Training set		Testing set		Whole data set	
		R^2	Root MSE	R^2	Root MSE	R^2	Root MSE
Growth traits and nitrogen metabolism							
Average daily gain, day 7–21	57.0–68.7 g/bird	0.92	0.56 g/bird	0.94	0.59 g/bird	0.93	0.57 g/bird
Average daily feed intake, day 7–21	72.7–82.9 g/bird	>0.99	0.06 g/bird	0.99	0.16 g/bird	>0.99	0.10 g/bird
Daily nitrogen intake, day 18–21	3.0–3.6 g/bird	0.45	0.09 g/bird	0.50	0.11 g/bird	0.46	0.10 g/bird
Daily nitrogen accretion, day 18–21	2.3–2.9 g/bird	0.36	0.09 g/bird	0.34	0.12 g/bird	0.35	0.10 g/bird
Plasma amino acids and biogenic amines, day 22							
Alanine	3.91–11.6 mg/100 ml	0.16	1.62 mg/100 ml	0.25	1.18 mg/100 ml	0.17	1.50 mg/100 ml
Allo–isoleucine[1]	0.04–0.78 mg/100 ml	0.11	0.13 mg/100 ml	0.17	0.12 mg/100 ml	0.12	0.13 mg/100 ml
α–amino–n–butyric acid[2]	0.05–0.80 mg/100 ml	0.60	0.04 mg/100 ml	0.15	0.12 mg/100 ml	0.30	0.07 mg/100 ml
Arginine	0.52–6.57 mg/100 ml	0.06	1.21 mg/100 ml	0.17	1.43 mg/100 ml	0.09	1.28 mg/100 ml
Asparagine	1.12–3.77 mg/100 ml	0.01	0.49 mg/100 ml	0.11	0.37 mg/100 ml	0.03	0.46 mg/100 ml
Aspartic acid	0.23–0.79 mg/100 ml	0.34	0.08 mg/100 ml	0.37	0.06 mg/100 ml	0.35	0.07 mg/100 ml
Citrulline[3]	0.07–0.78 mg/100 ml	–	–	–	–	–	–
Cysteine	0.51–1.41 mg/100 ml	0.31	0.17 mg/100 ml	0.35	0.15 mg/100 ml	0.32	0.16 mg/100 ml
γ–amino–n–butyric acid	0.15–1.25 mg/100 ml	0.20	0.21 mg/100 ml	0.21	0.20 mg/100 ml	0.20	0.21 mg/100 ml
Glutamine	9.15–29.0 mg/100 ml	0.10	2.97 mg/100 ml	0.11	2.04 mg/100 ml	0.10	2.72 mg/100 ml
Glutamic acid[4]	0.95–2.19 mg/100 ml	–	–	–	–	–	–
Histidine[4]	0.10–0.39 mg/100 ml	–	–	–	–	–	–
Isoleucine	0.26–1.47 mg/100 ml	0.09	0.23 mg/100 ml	0.11	0.22 mg/100 ml	0.09	0.23 mg/100 ml
Leucine	0.77–2.34 mg/100 ml	0.07	0.32 mg/100 ml	0.11	0.31 mg/100 ml	0.08	0.31 mg/100 ml
Lysine	0.56–5.21 mg/100 ml	0.12	0.83 mg/100 ml	0.14	0.86 mg/100 ml	0.13	0.84 mg/100 ml
Ornithine	0.09–1.06 mg/100 ml	0.08	0.13 mg/100 ml	0.15	0.20 mg/100 ml	0.09	0.15 mg/100 ml
Phenylalanine[4]	0.89–1.90 mg/100 ml	–	–	–	–	–	–
Phosphoethanolamine[5]	0.06–0.66 mg/100 ml	–	–	–	–	–	–
Phosphoserine	0.05–0.14 mg/100 ml	0.15	0.02 mg/100 ml	0.42	0.01 mg/100 ml	0.22	0.02 mg/100 ml
Proline	2.45–14.9 mg/100 ml	0.07	1.84 mg/100 ml	0.09	1.18 mg/100 ml	0.08	1.67 mg/100 ml
Serine	2.24–9.68 mg/100 ml	0.27	1.30 mg/100 ml	0.57	0.97 mg/100 ml	0.35	1.21 mg/100 ml
Taurine	0.66–2.77 mg/100 ml	0.08	0.38 mg/100 ml	0.18	0.49 mg/100 ml	0.10	0.42 mg/100 ml
Threonine	4.03–18.2 mg/100 ml	0.07	2.30 mg/100 ml	0.11	1.53 mg/100 ml	0.08	2.10 mg/100 ml
Tyrosine[4]	1.73–6.70 mg/100 ml	–	–	–	–	–	–
Valine	1.47–4.60 mg/100 ml	0.08	0.71 mg/100 ml	0.12	0.61 mg/100 ml	0.09	0.68 mg/100 ml
1 – Methylhistidine	0.07–0.20 mg/100 ml	0.07	0.02 mg/100 ml	0.14	0.02 mg/100 ml	0.08	0.02 mg/100 ml
β – Alanine	0.16–1.02 mg/100 ml	0.20	0.18 mg/100 ml	0.06	0.14 mg/100 ml	0.16	0.17 mg/100 ml

[1]50 observations set to 0 because no concentrations were detectable and lowest quantified concentration of 0.04 mg/100 ml was close to 0.

[2]4 observations set to 0 because no concentrations were detectable and lowest quantified concentration of 0.05 mg/100 ml was close to 0.

[3]Not statistically analyzed because concentrations in 28 samples were below the detection limit and lowest quantified concentration was not close to 0.

[4]Neural network did not converge because input and output variables did not correlate.

[5]Not statistically analyzed because concentrations in 3 samples were below the detection limit and lowest quantified concentration was not close to 0.

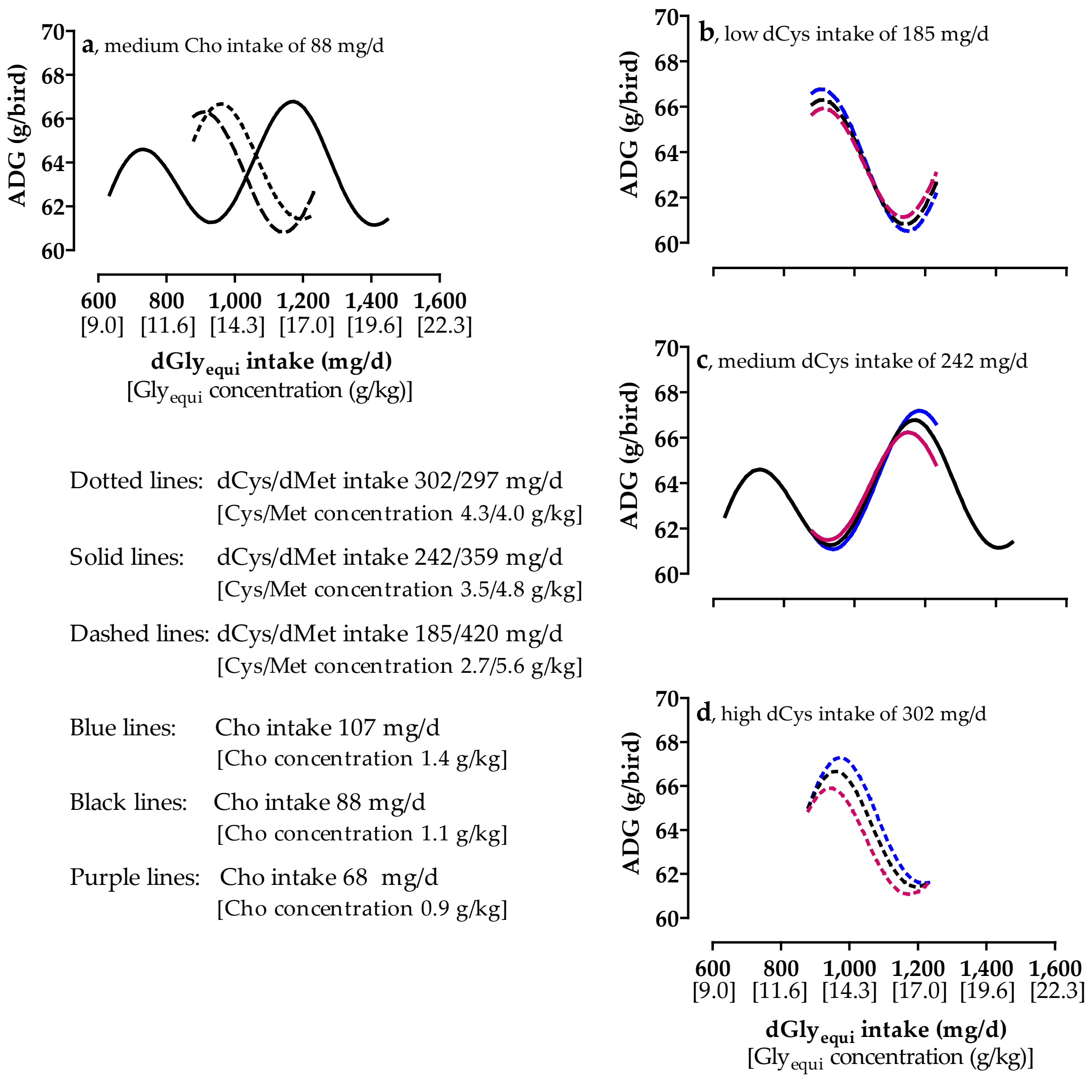

Figure S1. Effect of digestible glycine equivalent ($dGly_{equi}$) intake on average daily gain (ADG) of broiler chickens at varying digestible cysteine (dCys) and medium choline (Cho) intake from days 7 to 21 (**a**). The effect of $dGly_{equi}$ intake on ADG of broiler chickens at varying Cho and low, medium, and high dCys intake from days 7 to 21 (**b–d**). dCys and Cho intake levels correspond to level -1, 0, and +1 based on the fractional composite design.

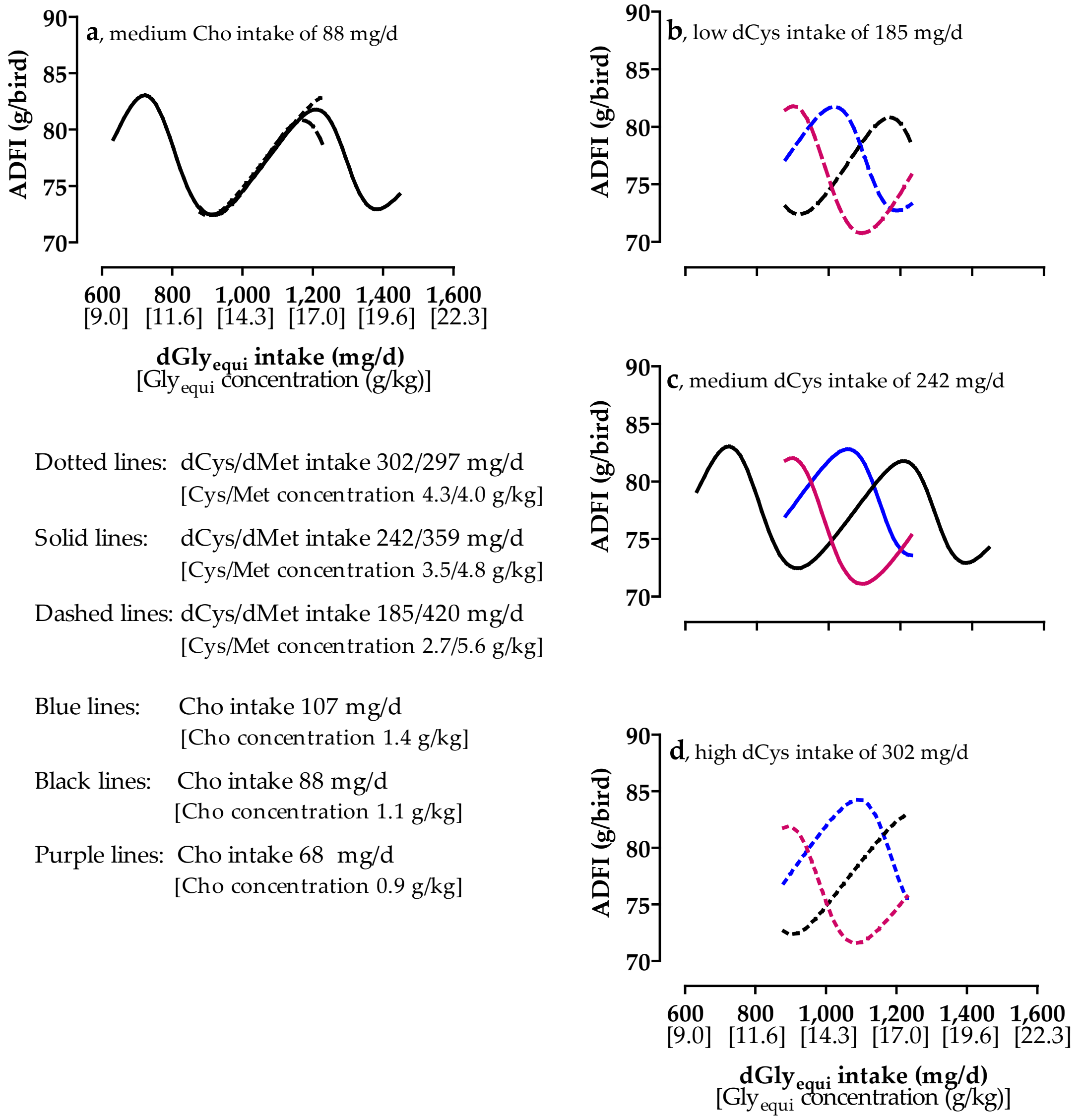

Figure S2. Effect of digestible glycine equivalent ($dGly_{equi}$) intake on average daily feed intake (ADFI) of broiler chickens at varying digestible cysteine (dCys) and medium choline (Cho) intake from days 7 to 21 (**a**) and effects of $dGly_{equi}$ intake on ADFI of broiler chickens at varying Cho and low, medium, and high dCys intake from days 7 to 21 (**b–d**). dCys and Cho intake levels correspond to level -1, 0, and +1 based on the fractional composite design.

References

1. WPSA - Working group No. 2 - Nutrition. The prediction of apparent metabolizable energy values for poultry in compound feeds. *Worlds Poult. Sci. J.* **1984**, *40*, 181–182.
2. Centraal Veevoederbureau. *CVB Feed Table 2016. Chemical composition and nutritional values of feedstuffs;* Federatie Nederlandse Diervoderketen: Rijswijk, the Netherlands, and Wageningen Livestock Research: Wageningen, the Netherlands, 2016.
